# The Influence of Sub-Zero Conditions on the Mechanical Properties of Polylactide-Based Composites

**DOI:** 10.3390/ma13245789

**Published:** 2020-12-18

**Authors:** Olga Mysiukiewicz, Mateusz Barczewski, Arkadiusz Kloziński

**Affiliations:** 1 Institute of Material Technology, Faculty of Mechanical Engineering, Poznan University of Technology, 61-138 Poznan, Poland; mateusz.barczewski@put.poznan.pl; 2 Institute of Chemical Technology and Engineering, Faculty of Chemical Technology, Poznan University of Technology, 61-138 Poznan, Poland; arkadiusz.klozinski@put.poznan.pl

**Keywords:** polylactide, waste filler, mechanical properties, sub-zero properties

## Abstract

Polylactide-based composites filled with waste fillers due to their sustainability are a subject of many current papers, in which their structural, mechanical, and thermal properties are evaluated. However, few studies focus on their behavior in low temperatures. In this paper, dynamic and quasi-static mechanical properties of polylactide-based composites filled with 10 wt% of linseed cake (a by-product of mechanical oil extraction from linseed) were evaluated at room temperature and at −40 °C by means of dynamic mechanical analysis (DMA), Charpy’s impact strength test and uniaxial tensile test. It was found that the effect of plasticization provided by the oil contained in the filler at room temperature is significantly reduced in sub-zero conditions due to solidification of the oil around −18 °C, as it was shown by differential scanning calorimetry (DSC) and DMA, but the overall mechanical performance of the polylactide-based composites was sufficient to enable their use in low-temperature applications.

## 1. Introduction

Polylactide or poly(lactic acid) (PLA) is an aliphatic polyester that can be synthesized from renewable sources, including corn starch [1,2,3]. Due to its relatively good availability and satisfactory mechanical properties, it is a common choice when an eco-friendly alternative to conventional polymeric materials is needed [4,5]. PLA can be processed using various technologies, including injection molding [3,6,7,8], extrusion [2,7,9] or fused deposition modeling 3D printing [10,11,12,13], and its properties can be tuned by different modifiers such as plasticizers [14,15,16,17], chain extenders [18] or nucleating agents [19,20,21]. Therefore, it can be successfully used in many applications, including the automotive industry, production of packages and disposable goods, or even special medical products [1,3,22,23,24]. Research works published in recent years, as well as industrial implementations, also indicate that polylactide can also be used as a matrix of composites. Even though conventional fillers such as glass fiber can be successfully embedded in PLA matrix [25], environmentally friendly fillers are the most common choice, as they allow us to obtain a fully bio-based biodegradable material. Numerous examples of polylactide-based composites reinforced with sisal fibers [26,27], flax fibers [28,29,30,31], wood [23,32] or hemp [33] can be found in the literature, but an even more sustainable solution is the application of the so-called waste fillers, i.e., the by-products mainly from agriculture or food industry such as nutshells, husks, seeds, waste fibers [9,34,35,36,37,38] and more. The application of waste fillers is not only in line with the idea of Circular Economy [5,39], but oftentimes improves different properties of the resulting composites [40,41]. In our previous works, we have shown that addition of linseed cake (i.e., the by-product of oil extraction from linseed or flax, *Linum usitatissimum* L.) to PLA causes an increase in crystallinity and elongation at break of the composites, which is attributed to the plasticizing effect of linseed oil contained by the filler [42,43]. It can be concluded that polylactide and its composites are a versatile group of environmentally friendly materials, which can be successfully used for different applications. According to the 2020 report by European Bioplastics, PLA makes up for 13.9% of 2.11 million tons of globally produced bioplastics and its production capacity is expected to grow significantly in the next years [44]. Therefore, a comprehensive analysis of polylactide properties is needed to fully benefit on its large-scale applications.

Like any other material, PLA has its disadvantages, such as brittleness and low crystallization rate [21]. However, from the application point of view, its thermomechanical stability at elevated temperatures is usually the main concern. Different strategies can be implemented to increase the thermomechanical stability of PLA, including the addition of mineral fillers [45,46] or application of nucleating agents [21,47]. A less researched area is the behavior of polylactide and its composites at sub-zero conditions. This situation is understandable as the glass transition temperature of PLA is around 50–80 °C [1] and its properties in the glassy state are considered stable. Nevertheless, as Kuciel et al. showed in their paper, the mechanical properties of polylactide-based composites change in sub-zero conditions [32]—a significant increase in tensile strength was denoted at −24 °C in comparison with its +24 °C value. It also needs to be realized that the addition of fillers or modifying agents to PLA can significantly alter its low-temperature behavior. The additives can significantly decrease the glass transition temperature of the matrix polymer or, in some cases, freeze or solidify in low temperatures [48]. The waste fillers, such as seed cake, can be very susceptible to the temperature changes due to the presence of the natural oil, which solidifies below 0 °C [49,50].

Even though room temperature properties are crucial in most applications, the sub-zero behavior of a material is also important, especially in the case of e.g., packages for frozen foods and other goods stored in low temperatures as well as outdoor applications in winter in colder climates. The temperature-induced embrittlement can result in a failure of a part, therefore the need for evaluation of the sub-zero behavior of materials is reasonable from both scientific and economic points of view.

The aim of this paper is a comprehensive analysis of sub-zero behavior of polylactide-based composites filled with linseed cake (LC). The dynamic and quasi-static mechanical properties of the composites with different LC grades were determined at room and sub-zero conditions and analyzed in relation to the phase transitions of the natural oil contained by the filler to describe the low temperature-induced changes of the material properties and to identify their causes. The temperature of −40 °C was chosen for the analysis, as it is slightly below the lowest temperatures denoted in Poland in last years, which is −35 °C [51]. Therefore, the paper will help to evaluate the performance of PLA-based composites in outdoor applications in Eastern European climate.

## 2. Materials and Methods

### 2.1. Materials

A multipurpose grade of polylactide Ingeo 2500 HP by Natureworks (Minnetonka, MN, USA), with Melt Flow Index of 8 g/10 min (210 °C, 2.16 kg), a density of 1.24 g/cm^3^ and d-isomer content <0.5%, was used as the matrix of the composites.

Linseed cake (LC) was obtained from a local Polish supplier (Laboratorium Biooil, Zielona Góra, Poland). To evaluate the influence of oil content on the composite’s properties, the LC was partially defatted to obtain 5 grades with 0.9, 4.6, 17.7, 30.4 and 39.8 wt% of natural oil, respectively. The defatting procedure consisted of mechanical mixing of the linseed cake with acetone, filtration and drying. The fillers were then screened through a 630 μm sieve. A more comprehensive description of the preparation of linseed cake with various oil content can be found in our previous study [43]. The natural oil extracted from linseed cake was also examined after removing the acetone by evaporation.

### 2.2. Sample Preparation

Composite samples with filler content fixed to 10 wt% were produces using a melt blending method and specimens for evaluation of mechanical properties were injection molded. First, the components were preliminarily mixed and dried overnight at 70 °C in a cabinet drier (Memmert, Schwabach, Germany). They were blended in a molten state using a Zamak EHD 16.2 co-rotating twin-screw extruder (Zamak Mercator, Skawina, Poland) operating at 120 rpm and 190 °C. The pelletized composites were dried as before and injection molded in a Battenfeld PLUS-35 machine (Heilbronn, Germany) with the following parameters: injection temperature of 210 °C, mold temperature of 50 °C, the injection pressure of 72 MPa. Pure PLA was processed along with its composites. The samples were named in reference to the used LC grade (for example, sample LC17.7 contains 10 wt% of linseed cake containing 17.7 wt% of natural oil). A detailed description of the linseed cake preparation and manufacturing of the composites can be found in our previous paper [43].

### 2.3. Methods

To evaluate the solidification of the oil extracted from the filler, its viscosity was evaluated using an oscillatory rotational rheometer Anton Paar MCR 301 (Anton Paar, Graz, Austria) in a 25 mm cone-plate configuration. The measurements were carried out in the temperature range from 30 to −80 °C with a cooling rate of 1 °C/min. A strain of 0.5% and a frequency of 10 Hz were applied.

The thermal properties of the linseed oil were analyzed by means of differential scanning calorimetry (DSC) using a Neztsch DSC 204 F1 apparatus (Netzsch, Selb, Germany). A sample of 10 mg was placed in a standard aluminum crucible (Netzsch, Selb, Germany) with pierced lid and cooled from 25 to −50 °C. It was held at −50 °C for 10 min and then heated to 200 °C. After that, the oil was cooled back to −50 °C. The measurements were performed with a heating/cooling rate of 10 °C/min in a Nitrogen atmosphere with a flow rate of 20 mL/min.

The dynamic mechanical properties of the composites and the pure PLA were assessed by Dynamic Mechanical Analysis (DMA, Anton Paar, Graz, Austria) in the oscillatory mode using an Anton Paar MCR 301 apparatus. The strain was fixed to 0.01% and the frequency was 1 Hz. The measurements were conducted in the temperature range from −80 to 25 °C.

The impact strength of notched samples was determined by Charpy’s method for two temperatures: −40 and 25 °C using a Ceast 9050 tester equipped with a 5 J pendulum. At least 5 samples of each type were tested. The differences between the mean values obtained by the composites tested in different conditions were evaluated by a one-way analysis of variance method (α = 0.05).

The tensile strength of the composites and pure PLA was evaluated in quasi-static conditions at 25 and −40 °C using a Zwick/Roell Z020 universal testing machine (Kennesaw, GA, USA). The crosshead speed was 2 mm/min during the tensile modulus evaluation and 50 mm/min during the remaining part of the test. At least 6 samples of each kind were tested. The differences between the mean values measured in different conditions were evaluated by one-way analysis of variance method (α = 0.05).

Brittleness *B* of the samples was evaluated according to the Equation (1) proposed by Brostow et al. [52], based on the results of the tensile test and DMA.

(1) $$B=\frac{1}{\varepsilon G^{\prime }}$$

where: *ε*—elongation at break determined in the quasi-static tensile test, *G′*—storage modulus evaluated by means of DMA.

## 3. Results

### 3.1. Linseed Oil Evaluation

**Figure 1:**
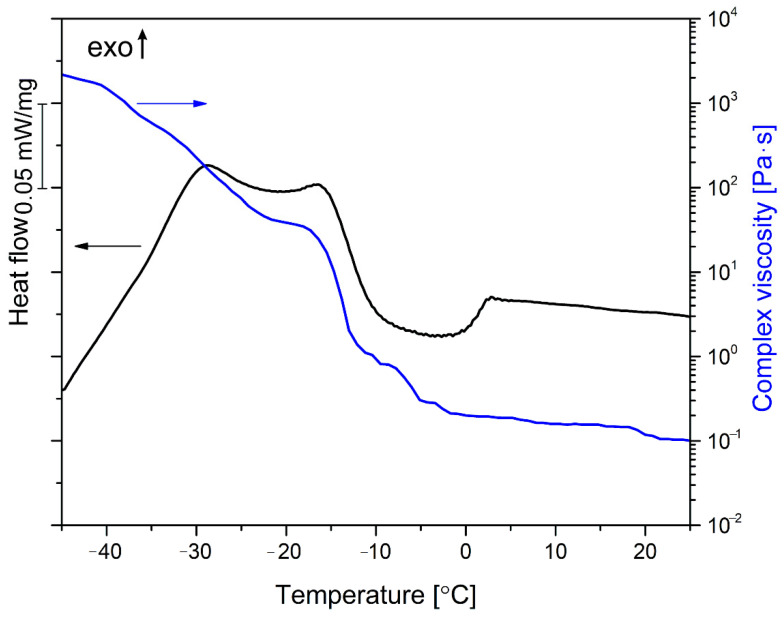
Changes of complex viscosity and the run of DSC curve for the linseed oil.

The changes of complex viscosity of linseed oil extracted from the filler in the function of temperature, as well as the DSC curve registered during cooling of the oil, are presented in Figure 1. The DSC thermogram shows two overlapping peaks at −16 °C and −29 °C. They indicate solidification (crystallization) of the vegetable oil [49]. The presence of the multiple crystallization peaks is typical for vegetable oil, which usually shows polymorphism and can create a hexagonal α form, an orthorhombic perpendicular β’ structure and a triclinic parallel β polymorph, each of them characterized with a different level of stability [50,53]. The crystalline form of the oil depends on solidification conditions as well as the amount of saturated and unsaturated fatty acids of different length [50]. The phase transition of natural oil is also shown by the changes in the complex viscosity, as presented in Figure 1. The η* value at room temperature is about 0.1 Pa·s, which is typical for vegetable oil [54]. Cooling to 0 °C results in only a slight increase in viscosity, which results from less intensive movements of the molecules [54]. After that, a drastic increase in complex viscosity takes place, which can be identified as the solidification of the oil. The highest rate of the change takes place in the temperature range of −11 to −17 °C, which overlaps with the slope of the crystallization peak recorded during the DSC measurements. According to the literature, linseed oil solidification takes place around −18 °C, which is consistent with the result of our experiment [55]. After that, the η* value stabilizes around 40 Pa·s and then increases again, which may result from the phase transition. The complex viscosity of linseed oil at −40 °C is 1640 Pa·s, which is 4 orders of magnitude higher than at room temperature. Based on those results, it can be predicted that the influence of linseed oil on the linseed cake-filled composites may be different in the room and sub-zero temperature ranges.

### 3.2. Evaluation of Polymeric Composites

#### 3.2.1. Dynamic Mechanical Analysis

**Figure 2:**
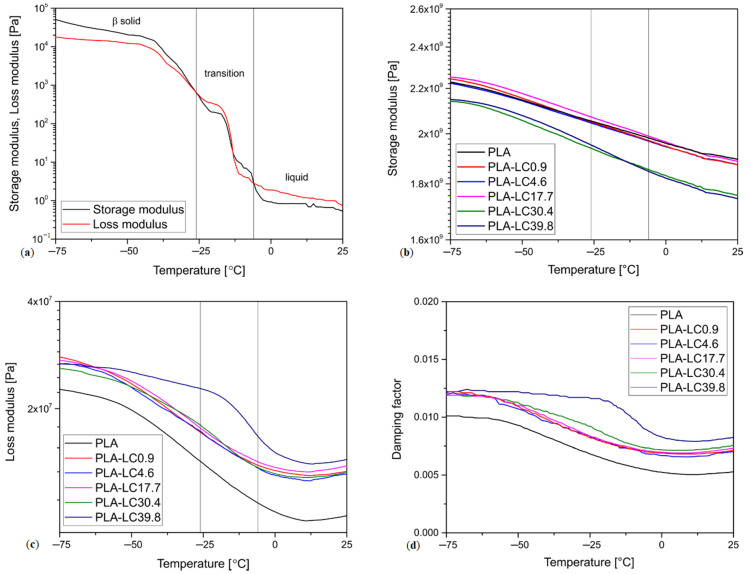
Changes of (**a**) storage and loss moduli of the linseed oil, determined by oscillatory rheology, (**b**) loss modulus of the polymeric samples (**c**) storage modulus and (**d**) damping factor of the polymeric samples in function of temperature, determined by DMA.

The run of the loss modulus of the linseed oil registered during the rheological measurements is shown in Figure 2a. The runs of storage and loss moduli (*G′* and *G”*, respectively) evaluated by DMA for the composite samples and the pure PLA vs. temperature are shown in Figure 2b,c. The run of the damping factor tanδ as a function of temperature evaluated by DMA for the composite samples is shown in Figure 2d.

When the value of *G′* is higher than *G”* the elastic properties of the material dominate, and it can be concluded that behaves as a viscoelastic solid. For *G′* < *G”* the viscous behavior is prevalent, and the sample can be considered a viscoelastic liquid. Therefore, the *G′* = *G″* crossover points can be associated with the phase transition of the oil [56]. As can be seen in Figure 1, below −26 °C linseed oil is in its solid form and above −6 °C it is liquid. In the range from −26 to −6 °C, it can be presumed that phase transitions between its polymorphs take place. The melting point of a fat-based substance can also be determined if a sudden drop of complex modulus below 100 Pa takes place [57]. In the case of linseed oil, this behavior takes place around −14 °C. Based on that information, it can be predicted that the properties of the linseed oil-modified polymeric composites can change due to phase transitions of the oil. The thermomechanical properties of the linseed cake-filled composites were evaluated by DMA and the resulting curves of *G′* and *G′* vs. temperature are presented in Figure 2b,c.

The storage modulus of the PLA and PLA-based composite samples does not change notably in the studied temperature range, but a steady decrease can be observed. The slope of the curve is steeper in the case of the PLA-LC-30.4 and PLA-LC-39.8 composites, which indicates that they are more prone to changes of properties in function of temperature. The oil-rich samples are also characterized by the lowest *G′* values, which can be associated with a lower amount of rigid lignocellulosic particles [58,59] and the fact that the linseed oil is softer than PLA, even in its solid form. The analysis of *G″* vs. temperature curve brings more insight into the thermomechanical properties of linseed cake-filled composites. The pure PLA and the linseed cake-based composites show a similar behavior: the values of *G″* decrease slowly in the function of temperature, which is a typical result for this polymer [60]. However, the changes in the loss modulus in the function of temperature are not the same for all the studied polymers. The unfilled PLA and its composites containing up to 30.4 wt% of oil in the filler present a similar, steady decrease in *G′*, whereas the loss modulus of the PLA-LC-39.4 sample remains stable up to −25 °C and then decreases rapidly. Interestingly, this sudden change of *G′* takes place in the same temperature range as the phase transition of the natural oil contained by the sample. Therefore, it can be concluded that it is melting of the linseed oil contained by the filler, which causes a distinct change in loss modulus of the composite. The decrease in the oil’s *G″* results in a lowering of the composite’s loss modulus, which indicates that the influence of this modifying agent on the PLA-based material is notable. The fact that the relaxation of the oil contained by the composite can be identified in its *G″* plot in the same temperature range as in the case of the rheological measurements indicates that PLA and linseed oil are not well miscible [61]. A similar result was denoted during observations by scanning electron microscopy of the linseed cake-filled samples, as it is described in our previous research [43].

Interestingly, the melting of the oil can be also observed on the tan δ curve presented in Figure 2d. In this case, a deviation from the linear shape of the damping factor plot can be also spotted for the PLA-LC-30.4 composite. Nevertheless, due to small values of tan δ in this temperature range, the signal is noisy; therefore, it can be decided that the run of the *G″* curve is a more reliable indicator of oil relaxation in the PLA-LC composites.

#### 3.2.2. Impact Strength Evaluation

The mean values of impact strength evaluated in room temperature and at −40 °C along with *p*-values obtained in one-way analysis of variance, indicating the presence of significant differences between the dynamic mechanical properties of the samples tested in different conditions are collected in Table 1. The impact strength of pure PLA measured at 25 °C is about 2.4 kJ/m^2^ and, due to the addition of the low-fat linseed cake, it decreases (for the PLA-LC0.9 and PLA-LC4.6 samples) or remains at the same level (in the case of PLA-LC17.7 and PLA-LC30.4 ones). This result is typical in the case of polymeric composites containing low aspect ratio filler, whose particles act as points of stress concentration and facilitate the propagation of cracks [62]. Similar behavior was denoted by Andrzejewski et al. in the case of polycarbonate filled with biochar, whose Izod impact strength dropped from 650 J/m to 13 J/m [63]. However, the application of the oil-rich LC, such as LC39.8, causes an increase in impact strength. This result can be attributed to the plasticizing effect of the natural oil, which promotes the movements of macromolecules. Consequently, the material can deform during the impact and dissipates more energy. It can be observed that the plasticizing effect of the natural oil compensates for the decrease in impact strength due to the presence of rigid lignocellulosic particles.

The decrease in the testing temperature to −40 °C causes a change in the impact strength for all the tested materials, but no relationship between the low and room temperature values can be observed. What is more, as indicated by the *p*-values > 0.05, the difference is statistically insignificant for all the samples except for the PLA-LC39.8. In the case of the latter, the impact strength tested at −40 °C is 0.77 kJ/m^2^ lower in comparison with the result of the room temperature measurement. This effect can be explained by solidification of the natural oil, whose molecules lose the abilities to move and thus promote the movements of PLA macromolecules. Even though the oil solidification of oil takes place in all the linseed cake-modified samples, only in the case of PLA-LC39.8 its content is high enough to cause a significant difference.

#### 3.2.3. Tensile Strength Evaluation

**Figure 3:**
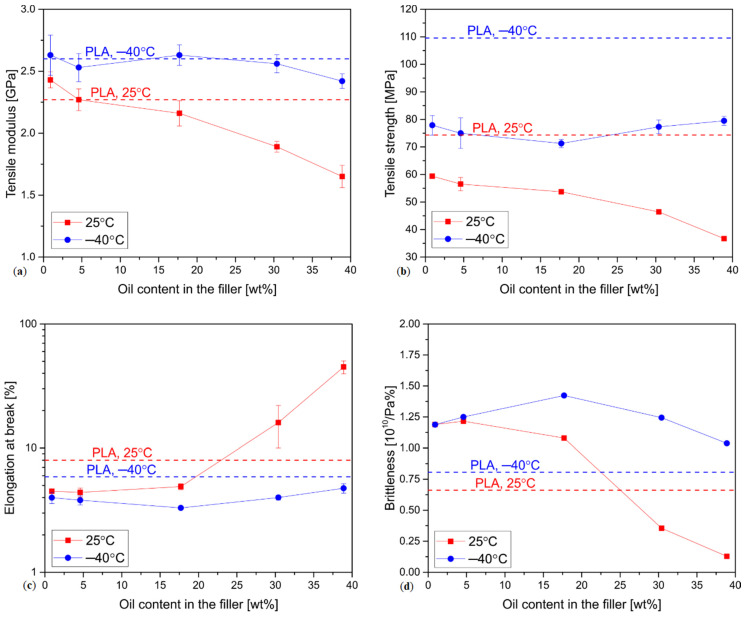
Tensile properties of the composites and pure PLA tested at different temperatures in function of oil content in the fillers, (**a**) tensile modulus; (**b**) tensile strength; (**c**) elongation at break; (**d**) brittleness. The values obtained for pure polylactide are indicated by the dashed line.

The changes of tensile modulus E, tensile strength Rm, elongation at break *ε* and brittleness *B* of the samples evaluated in different conditions are presented in the function of oil content in the filler in Figure 3. The difference between the corresponding samples of each kind tested at −40 °C and 25 °C was statistically significant (*p*-values < 0.05).

The tensile modulus of the unfilled PLA tested at room temperature is 2.26 GPa. The addition of 10% of the defatted linseed cake results in a small growth of the E value, but increasing the oil content in the filler causes a steady decrease in Young′s modulus, which indicates the plasticizing effect of LC on PLA. The PLA-LC39.8 sample shows the E value of about 1 GPa lower than the neat polylactide. The decrease in the testing temperature to −40 °C results in a significant increase in the tensile modulus for all the samples. Its value for pure PLA increases from 2.26 GPa to 2.60 GPa. This behavior is common for the thermoplastic polymers and it can be explained by a decrease in the distance between the molecules and a resulting increase in the binding forces [64]. Even though the Young’s modulus of the composite samples tested at −40 °C is different from the one of the unfilled polymer, only in the case of the sample with the highest oil content, the difference is statistically significant (*p* < 0.05). Therefore, the plasticizing effect of linseed cake is notably reduced. This behavior can be attributed to the solidification of the oil (as shown by the rheological measurements), which can no longer facilitate the movements of the macromolecules. This unwanted effect of solidification is, in fact, a common problem among the plasticizers [48]. Nevertheless, the composite containing the highest content of linseed oil presents a significantly lower E value. This behavior is presumably due to a replacement of a part of the rigid polymer with considerably softer solidified linseed oil. It also needs to be noticed that a similar situation was observed in the case of the storage modulus of the PLA and PLA-LC-39.8 samples tested at −40 °C.

The tensile strength of the pure PLA at room temperature is 74.3 MPa, which is a common value for this polymer [1]. The linseed cake-filled composites show considerably lower Rm values, which decrease with the linseed oil content. This is typical to polylactide plasticized with non-epoxidized oils, which lack epoxy groups capable of reacting with the polymeric chains [26]. What is more, the linseed oil does not mix well with PLA, creating separate domains [43], so the limited interactions of the matrix and the plasticizer also reduce the tensile strength of the composite [65,66]. The tensile strength of PLA tested at −40 °C is 110 MPa. Similarly to the increase in Young′s modulus, the enhancement of Rm at sub-zero temperatures can be explained by the intensification of the interactions between the polymeric chains, which come into close proximity with one another [67]. The composites show tensile strength −40 °C lower than the pure polymer but still higher in comparison to the results achieved at room temperature. No clear relationship between the Rm and oil content can be distinguished, which can indicate that oil solidification improves its interactions with PLA, presumably due to the differences in thermal expansion coefficients and mechanical interlocking of the solidified oil particles and the polymeric matrix. Therefore, the tensile strength of the composites is mostly influenced by the filler dispersion and structural flaws such as porosities created in the injection molding process.

The changes of elongation at the break due to the addition of oil-rich linseed cake were considered one of the main arguments to support the hypothesis of the plasticizing influence of this filler in our previous research [42], therefore its change should be observed due to oil solidification. The results of the tensile test confirm this prediction. At room temperature pure PLA shows *ε* of 8%. The addition of the defatted linseed cake causes a decrease in elongation to about 4.5–5.0%, which is a typical effect of lignocellulosic particle-like filler. When the oil content in the filler exceeds 30%, the ε increases to 45%. The linseed oil acts as an internal lubricant for PLA and enables it to deform before fracture. Completely different behavior can be observed in the case of the LC-filled composites tested at −40 °C. The values of elongation at break of the pure PLA and its composites are lower in comparison to the room temperature results. This result is typical for thermoplastic composites and it is commonly explained by reduced molecular mobility of the polymer at low temperatures [67]. What is more interesting, the elongation at break of the composites no longer depends on the oil content in the filler—the *ε* value of the PLA-LC-39.8 sample decreases from 45% at 24 °C to 4.8% at −40 °C. Therefore, the reduction of the plasticizing effect of the linseed cake not only influences the tensile modulus and tensile strength of the composites but, even more notably, their elongation at break.

Brittleness, as proposed by Brostow et al., depends on both dynamic (storage modulus) and quasi-static mechanical (elongation at break) properties of a material [52,68]. The higher its value, the more brittle (i.e., less ductile, more prone to cracking) is a material. The PLA-based composites tested at room temperature initially show higher brittleness, but its values decrease with oil content, proving its plasticizing influence. In the case of the sub-zero measurements, the values denoted for the composites containing the defatted linseed cake are almost the same as in the case of the room temperature testing. The difference gets more and more visible as the oil content increases—the brittleness of the oil-rich samples does not decrease as the solid oil does not have the same modifying properties as the liquid one.

## 4. Conclusions

Both the sub-zero and room temperature mechanical properties of polylactide and its composites filled with linseed cake were tested. It was found that tensile modulus and tensile strength of the studied materials notably increase in low temperatures—the E values changed from 1.65–2.43 GPa at 25 °C to 2.42–2.63 GPa at −40 °C. This growth was associated with intensification of the interactions of the macromolecules at low temperatures. The impact strength of the PLA and most of its LC-filled composites did not change significantly except for the PLA-LC39.8 sample, whose impact strength decreased significantly. Even though all the studied materials showed lower elongation values at −40 °C than at room temperature, the decrease was especially notable for the composites with the highest oil content. This behavior was attributed to the solidification of the linseed oil around −18 °C, as it was shown in by DSC and DMA. It was found that even though the plasticizing effect of linseed oil is highly reduced due to its phase transition, the LC-filled polylactide composites present good mechanical properties at −40 °C and therefore can be successfully used in sub-zero applications, especially if strength and rigidity are needed.

## Figures and Tables

**Table 1:** The impact strength of the samples tested at different temperatures.

Impact Strength [kJ/m^2^]	PLA	PLA-LC0.9	PLA-LC4.6	PLA-LC17.7	PLA-LC30.4	PLA-LC39.8
25 °C	2.38 ± 0.2	1.83 ± 0.3	2.17 ± 0.4	2.34 ± 0.4	2.42 ± 0.4	3.12 ± 0.1
−40 °C	2.24 ± 0.1	1.95 ± 0.3	2.19 ± 0.1	2.18 ± 0.1	2.63 ± 0.3	2.35 ± 0.2
*p*-value	0.2617	0.6078	0.9448	0.4587	0.4115	0.0002
